# Robot-assisted kidney transplantation: a propensity score-matched cohort analysis of early experience

**DOI:** 10.1097/JS9.0000000000002019

**Published:** 2024-08-05

**Authors:** Seung J. J. Kim, Sangwan Kim, Ara Cho, Ahram Han, Jongwon Ha, Sangil Min

**Affiliations:** a Department of Surgery, Seoul National University College of Medicine; b Institute of Health Policy and Management, Seoul National University Medical Research Center; c Transplantation Research Institute, Seoul National University College of Medicine

**Keywords:** kidney transplantation, minimally invasive surgery, robotic assisted kidney transplantation

## Abstract

**Background:**

Kidney transplantation is the preferred treatment for patients with end-stage kidney disease. Since the introduction of robot-assisted kidney transplantation (RAKT), several centers have applied this technique as an alternative to open kidney transplantation (OKT). The objective of this study is to analyze our early experience, focusing on surgical technique and learning curve, and postoperative outcomes of RAKT.

**Methods:**

The authors retrospectively reviewed 782 living donor kidney transplantation recipients between January 2018 and January 2024. A propensity score-matched cohort of 50 RAKT and 150 OKT patients was evaluated for intraoperative and postoperative variables. Shewhart control charts and CUSUM analysis were used to evaluate the technical outcomes and learning curves of RAKT. Postoperative eGFR values, complications, and biopsy results were compared for overall graft function and safety.

**Results:**

RAKT patients were associated with significantly longer overall operative, rewarming, and anastomosis times. Although overall postoperative eGFR trends showed comparable graft function between RAKT and OKT recipients (51.35±2.64 vs. 54.01±1.45; *P*=0.315), RAKT patients with extremely long rewarming times exhibited aggravated chronic scores at 1-year protocol biopsies (Δ Chronicity Index=4.45±1.92, *P*<0.001). CUSUM analysis of rewarming time revealed that proficiency in RAKT is achieved after ~15 cases.

**Conclusions:**

Despite longer anastomosis and ischemic times, even during the early stages of RAKT adoption, the RAKT group did not differ significantly in graft function or postoperative complications from the OKT group.

## Introduction

Highlights

- A retrospective review of 782 living donor kidney transplant recipients was done. A propensity score-matched cohort of 50 robot-assisted kidney transplantation and 150 open kidney transplantation patients was evaluated for intraoperative and postoperative variables.
- A step-by-step review of our surgical technique was detailed.
- RAKT patients were associated with significantly longer total operation time, in addition to rewarming time, nonanastomotic time during rewarming time, venous anastomosis time, and arterial anastomosis time.
- CUSUM analysis revealed rewarming time learning curves to be ~15 cases. Vascular anastomosis learning curves required 5−10 cases, but nonanastomotic time learning curves have yet to be overcome during our study period.
- Evaluation of postoperative graft function at POD 0, POD 5, POD 7, POD 1 month, POD 3 months, POD 6 months revealed comparable result between RAKT and OKT groups. Further analysis of RAKT risk groups based on RAKT rewarming times also revealed no significant differences in graft function.
- Postoperative complications, including slow graft function, delayed graft function, graft survival, and patient survival, were comparable across RAKT and OKT groups.
- Postoperative protocol biopsy evaluation showed aggravated Chronicity Index scores at POD 1-year biopsies in RAKT patients. Significant greater increase in chronic scores of RAKT patients with longer rewarming times may indicate chronic histological damage is associated with longer rewarming times in RAKT.

Kidney transplantation is the preferred treatment for patients with end-stage renal disease^1,2^. Traditional open kidney transplantation (OKT) has long been the standard approach, but morbidities associated with open surgery, such as postoperative pain, prolonged hospital stay, and higher risk of surgical site infections, has been a subject of concern. The adoption of minimally invasive surgery (MIS) across many surgical specialties has enabled surgeons to mitigate these issues^3,4^. Robotic tools, with their enhanced precision and dexterity, have provided significant advantages in reducing postoperative complications and improving patient outcomes^5–7^.

Since the introduction of the first fully robotic-assisted kidney transplantation (RAKT) by Giulinotti *et al*.^8^ at the University of Chicago in 2009, a number of North American and European centers have applied this technique as an alternative to OKT^9–11^. Recently, the first robotic kidney transplantations in South Korea was reported by Kim *et al*.^12^ in 2019, and the technique has seen greater adoption across Asia, reflecting a greater interest in advancing this minimally invasive approach^13,14^. Although RAKT is associated with longer ischemic and anastomosis times, early studies have demonstrated its feasibility and safety^15–20^.

Despite the standardization of RAKT’s surgical technique, it remains a complex procedure, and the literature currently lacks comprehensive studies on its early experience. Studies have shown surgeons with prior MIS experience quickly adapt to RAKT, overcoming the learning curve and achieving satisfactory outcomes^21–23^. However, the early experience for surgeons without previous MIS experience is less understood.

This study aims to comprehensively analyze our early experience of RAKT, and compare the outcomes with those of OKT. We provide a detailed description of our surgical technique for RAKT, highlighting the challenges encountered and the subsequent refinements made to enhance our procedure. Operative variables were evaluated, and a cumulative sum (CUSUM) analysis was conducted for each individual surgeon to evaluate the learning curve associated with RAKT. Furthermore, we compared postoperative graft function, complications, and biopsy scores to comprehensively assess the overall outcomes and safety of RAKT.

## Methods

### Study design

This retrospective study evaluates patients who underwent living donor kidney transplantation (LDKT) at Seoul National University Hospital (SNUH) between 1 January 2018 and 31 January 2024. The initial cohort comprised 782 patients: 50 received RAKT and 732 received OKT. To ensure a consistent adult cohort and an accurate comparison of intraoperative and postoperative outcomes, patients with missing operative data, those under 16 years of age, and recipients of simultaneous multiorgan transplants were excluded.

The procedures were performed by three surgeons, all experienced in OKT (surgeon A >1000, surgeon B >2000, surgeon C >500), but without prior laparoscopic or robotic surgery experience. They invested a significant amount of time in training and familiarizing themselves with the robotic program. They attended biweekly sessions for 6 months at Intuitive Surgical’s Center for Surgical Innovation at Sangam DMC High-Tech Industry Center, and completed its technology training program. Furthermore, surgeons practiced vascular anastomosis on robotic simulators, suturing balloons or gloves in an end-to-end or end-to-side continuous manner. Prior to the initial case, multiple planning meetings involving operating surgeons, assistant fellows, surgical assistant nurses, and scrub nurses were held to comprehensively review the procedure and confirm equipment availability.

Demographics were collected, and to mitigate potential confounding factors, propensity score analysis, then 1:3 matching was conducted. A matched cohort of 200 patients (50 RAKT, 150 OKT) was analyzed for intraoperative and postoperative outcomes.

The study was reported in line with the STROCSS criteria, approved by the SNUH Institutional Review Board (IRB No 2305-119-1434), and registered on clinicaltrials.gov (NCT)^24^.

### Surgical technique

Our approach aligns closely with previously established techniques, with the distinction of our Pfannenstiel incision for graft introduction and open to ureteroneocystostomy^25,26^. Perioperative antibiotics, specifically cefazolin, was administered preoperatively and continued until postoperative day 3. All surgeries were conducted using the Da Vinci Xi Robotic Surgical system.

#### Bench work

All kidney grafts were provided by living donation, with simultaneous graft retrieval performed via hand-assisted laparoscopic donor nephrectomy. Following retrieval, grafts are perfused using cold histidine-tryptophan-ketoglutarate solution. Graft vessels are carefully dissected and double-J stents are inserted in the ureter depending on surgeon preference.

**Figure 1:**
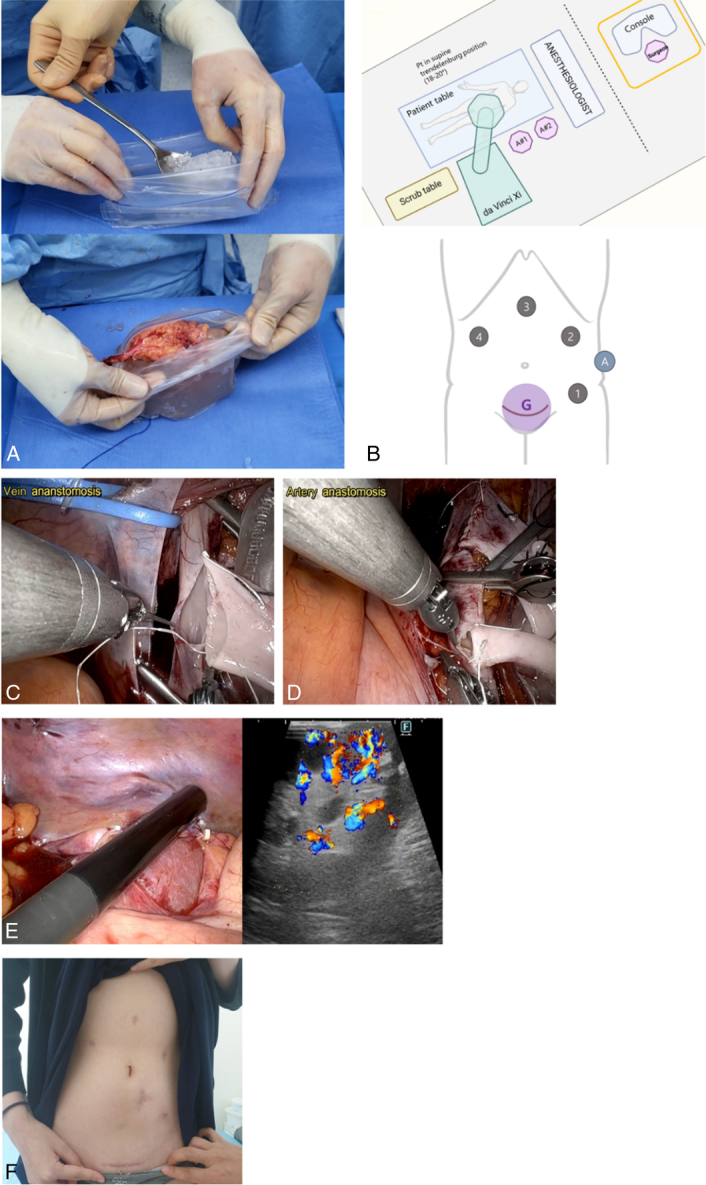
Surgical procedure of robotic assisted kidney transplantation. (A) Bench work and laparoscopic bag for cooling, (B) Patient positioning and trocar positioning, (C) Venous anastomosis, (D) Arterial anastomosis, (E) Intraoperative Ultrasound Doppler, (F) Postoperative scar. Camera port at arm 2, working ports at arm 1, 3, and 4, assistant port at the left flank, and a GelPort system is inserted through the Pfannenstiel incision. Instrument selection during operative bed preparation: Maryland bipolar forceps in arm 1, monopolar curved scissors in arm 3, ProGrasp forceps in arm 4. Instrument selection during vascular anastomosis: Black Diamond microforceps at arm 1, large needle driver and Potts scissors (for venotomy and arteriotomy) at arm 3, and ProGrasp forceps at arm 4.

To maintain regional hypothermia, grafts are placed in a laparoscopic bag filled with ice slush, with an opening allowing for vascular anastomosis (Fig. 1A).

#### Patient and trocar positioning

With the patient in supine position, an 8 mm robotic port is first introduced at the epigastric region for pneumoperitoneum. Three additional 8 mm robotic ports are inserted at the left lower, left upper, and right upper quadrant regions, and one 12 mm assistant port is positioned at the left flank (Fig. 1B). Following the placement of robotic and assistant ports, the patient is positioned to 18–20° Trendelenburg position for in preparation for docking.

#### Robotic phase I (operative bed preparation)

The robotic system is docked at the patient’s left lower side, with assistants stationed to the right and the scrub table to the left (Fig. 1B). With the right external iliac vessels identified and fully skeletonized, the external iliac artery (EIA), and external iliac vein (EIV) are looped with elastic vessel loops for easy manipulation during anastomosis. A peritoneal flap is prepared laterally for graft reperitonealization, and a tunnel is made medially for the ureter.

#### Incision and graft introduction

The patient is positioned back to supine position for graft introduction. For graft access, a Pfannenstiel incision is done, followed by GelPort system insertion. Abundant ice slush is first inserted, then the prepared graft is inserted into the abdominal cavity. After graft introduction, the patient is returned to Trendelenburg position for the second robotic phase.

#### Robotic phase II (vessel anastomosis and reperfusion)

The robotic system is docked in the same position as before, and the graft is positioned laterally to the external iliac vessels. The EIV is clamped using bulldog clamps and venotomy is performed. The graft renal vein is anastomosed by end-to-side continuous technique using Gore-Tex CV-6 suture (Fig. 1C). Arterial anastomosis is done in the same manner as venous anastomosis (Fig. 1D). Once vascular anastomosis is completed and reperfusion is visually confirmed, the graft is reperitonealized using hemolock clips, and the ureter is placed through the previously created tunnel.

Of note, even while avoiding patients with calcified proximal EIA through preoperative CT angiography evaluation, we struggled with uncontrolled iliac artery bleeding during our early cases. A change to 350 g closing pressure bulldog clamps, from the initial 225 g closing pressure clamps, greatly improved our ability to control EIA bleeding.

#### Ureteroneocystostomy and wound closure

Once the robotic system is undocked, the patient is placed back into supine. The GelPort is removed and open ureteroneocystostomy is performed according to the Lich–Gregoir technique. After confirming urination, final laparoscopic re-evaluation and Jackson–Pratt drain insertion is done. Intraoperative Doppler ultrasound assessment can be performed at this point for final evaluation of graft perfusion (Fig. 1E). Postoperative wound is shown in Figure 1F.

### Immunosuppression

Immunosuppressive therapy for both groups consisted of the same induction therapy and triple maintenance agents. Induction therapy included basiliximab or rabbit antihuman thymocyte immunoglobulin. Routine maintenance therapy consisted of tacrolimus, mycophenolate mofetil, and steroids.

Tacrolimus was initiated within 24 h before transplantation at a dose of 0.075–0.1 mg/kg twice daily. Tacrolimus dosage was adjusted to achieve target trough concentration levels of 8–10 ng/ml during the first 3 months, then 6–8 ng/ml from 3 to 6 months. Mycophenolate mofetil, or an equivalent dose of mycophenolic acid, was administered at a fixed dose of 500 mg twice daily. An initial 500 mg intravenous bolus of methylprednisolone was administered intraoperatively, then rapidly tapered over 4 weeks to a maintenance dose of 5 mg oral prednisolone.

### Study variables

The following demographic variables were included: ABO incompatibility (ABOi), Donor Specific Antibody (DSA) positivity, preoperative desensitization (plasma exchange, IVIG, rituximab), number of HLA mismatches, age, sex, weight, height, BMI, history of diabetes mellitus, hemodialysis status, dialysis vintage, donor age, and graft weight.

Operative variables assessed were operative time, console time, incidence of open conversion, estimated blood loss, warm ischemic time (WIT), cold ischemic time (CIT), rewarming time (RT), nonanastomotic time (NAT) during RT, venous anastomosis time, arterial anastomosis time, and postoperative day (POD) of discharge. WIT (donor artery clamp to perfusion start), CIT (perfusion start to graft introduction), RT (graft introduction to reperfusion), and NAT (rewarming time excluding venous and arterial anastomosis time) were defined as such.

Postoperative outcomes evaluated included eGFR (CKD-EPI-Cr formula, ml/min/1.73 m^2^) levels at POD 0, POD 5, POD 7, 1 month, 3 months, and 6 months^27^. Slow graft function (SGF), delayed graft function (DGF), graft survival, and patient survival were also assessed. SGF was defined as Cr >3 mg/dl on POD 5, and DGF as the need for dialysis before POD 7. Graft and patient survival were assessed with a 6-month follow-up.

Routine postoperative biopsies, performed at approximately POD 10 days and POD 1-year, were assessed and graded according to Banff 2017 or 2019 criteria^28,29^. Chronicity Index was derived from the sum of interstitial fibrosis (ci), tubular atrophy (ct), arterial intimal fibrosis (cv), and chronic glomerulopathy (cg, score doubled) scores, with a maximum score of 15^30^.

### Statistical evaluation

Frequencies and percentages were used to describe categorical variables; mean and SD or median and interquartile ranges were reported for continuous variables. Mann–Whitney or Student’s *T*-tests for quantitative, and *χ* ^2^ test for qualitative variables, were applied to compare the distribution of variables between groups.

**Figure 2:**
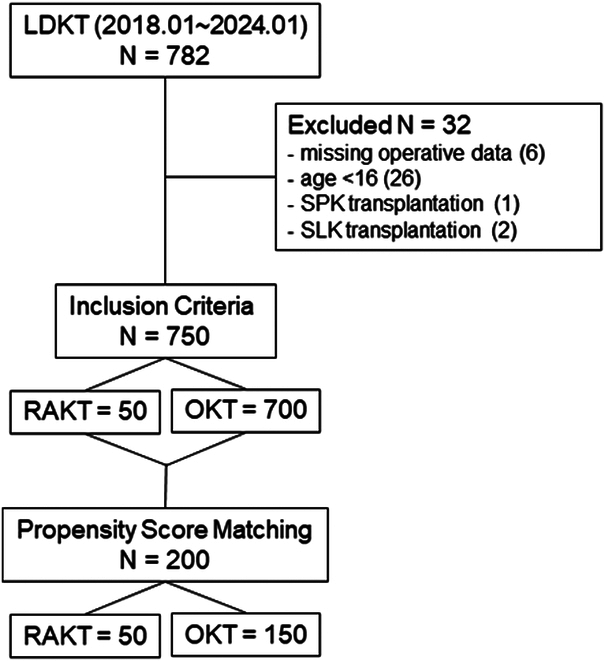
Consort diagram for patient selection. LDKT, living donor kidney transplantation; OKT, open kidney transplantation; RAKT, robotic assisted kidney transplantation; SPK, simultaneous pancreas kidney, SLK, simultaneous liver kidney.

For propensity score analysis, multiple logistic regression analysis was done with RAKT and OKT as binary dependent variables, and the following variables as independent variables: preoperative desensitization, number of HLA mismatches, age, sex, BMI, history of DM, hemodialysis, dialysis vintage, donor age, graft weight. Subsequently, a 1:3 nearest-neighbor match was performed, resulting in 50 RAKT and 150 OKT patients (Fig. 2).

Technical outcomes and learning curves of RAKT for individual surgeons were assessed using Shewhart control charts and CUSUM analyses of RT, NAT, venous anastomosis, and arterial anastomosis times. As OKT is the gold standard for kidney transplantation, mean times of each propensity score-matched OKT case were used as reference. For Shewhart control charts, respective OKT mean times were set as the central line, mean +1SD as the upper cutoff of the target range, and mean +2SD as the alert threshold. For CUSUM analysis, the target value was also set as corresponding OKT mean +1SD times, with cumulative difference of observed values for each case plotted over time. The learning curve was deemed complete once CUSUM graph curves reached a plateau.

A linear mixed-effects model was employed to assess the effects of robotic surgery at various postoperative day points. Fixed effects of the postoperative day, the presence of robotic surgery, and their interaction were first considered. The initial model is as follows:


$${\mathrm{eGRF}}_{\mathrm{ij}}=\beta_{0}+\beta_{1}\mathrm{PO}D_{\mathrm{ij}}+\beta_{2}\mathrm{Robo}t_{i}+\beta_{3}\mathrm{PO}D_{\mathrm{ij}}\times \mathrm{Robo}t_{i}+u_{i}+\epsilon_{\mathrm{ij}}$$

$\beta_{1}$ represents the change in eGFR over time, $\beta_{2}$ represents a change in intercept due to robotic surgery, and $\beta_{3}$ represents a change in slope due to robotic surgery. The interaction of postoperative day and surgery type was not significant, as the slope remained constant regardless of operation type. The interaction was removed, and the final model is as follows:


$${\mathrm{eGRF}}_{\mathrm{ij}}=\beta_{0}+\beta_{1}\mathrm{PO}D_{\mathrm{ij}}+\beta_{2}\mathrm{Robo}t_{i}+u_{i}+\epsilon_{\mathrm{ij}}$$

Furthermore, to assess the impact of RT on the graft function of RAKT patients, they were divided based on OKT RT mean and SD times: moderate risk (<mean +2SD), high risk (mean +2SD~3SD), and very high risk (>mean +3SD). The difference between individual RAKT and matched OKT patient eGFR values were calculated, then compared across each follow-up period. Additionally, overall eGFR trends were analyzed between risk groups through mixed model analysis.

Python 3.12 and R 4.3.2 were used for statistical analysis. Statistical significance was defined as *P*<0.05.

## Results

### Demographics

During the study period, a total of 782 patients (50 RAKT and 732 OKT) underwent LDKT. Of these, 750 patients met the inclusion criteria. Patients’ demographics before and after propensity score matching are detailed in Table 1. Matching resulted in a significant reduction of standardized mean differences (SMDs) for most covariates, with no significant differences observed across all variables, indicating a high level of comparability. All subsequent analyses were performed on the propensity score-matched cohort.

**Table 1:** Baseline characteristics of patients before and after propensity score matching.

	Before matching				Post matching			
	RAKT (*n*=50)	OKT (*n*=700)	SMD	*P*	RAKT (*n*=50)	OKT (*n*=150)	SMD	*P*
ABOi, *n* (%)	16 (32.0)	211 (30.1)	0.040	0.907	16 (32.0)	37 (24.7)	0.163	0.405
DSA+, *n* (%)	3 (6.0)	68 (9.7)	−0.138	0.537	3 (6.00)	16 (10.7)	−0.169	0.486
Desensitization, *n* (%)	17 (34.0)	250 (35.7)	−0.036	0.927	17 (34.0)	47 (31.3)	0.057	0.861
HLA mismatch count, n±SD	3.64±1.66	4.54±2.07	−0.481	<0.001	3.64±1.66	3.65±2.05	−0.007	0.866
Recipient age, years±SD	38.08±13.52	49.99±13.30	−0.888	<0.001	38.08±13.52	40.93±14.38	−0.204	0.231
Sex, M:F, *n* (%)	19 (38.0): 31 (62.0)	435 (62.1): 265 (37.9)	−0.498	0.001	19 (38.0): 31 (62.0)	66 (44.0): 84 (56.0)	−0.122	0.563
Weight, kg±SD	60.38±13.08	63.21±12.25	−0.223	0.061	60.38±13.08	60.23±12.15	0.012	0.791
Height, cm±SD	163.75±9.63	164.75±8.68	−0.108	0.401	163.75±9.63	164.51±8.28	−0.084	0.653
BMI, kg/m^2^±SD	22.41±3.82	23.21±3.66	−0.214	0.055	22.41±3.82	22.22±3.58	0.049	0.972
DM, *n* (%)	12 (24.0)	240 (34.3)	−0.228	0.183	12 (24.0)	39 (26.0)	−0.046	0.925
Dialysis, *n* (%)	34 (68.0)	470 (67.1)	0.018	1.000	34 (68.0)	95 (63.3)	0.098	0.670
Dialysis vintage, days (IQR)	191.5 (78.25–363.75)	292.0 (126.0–860.0)	−0.416	0.012	191.5 (78.25–363.75)	184.0 (106.0–404.0)	−0.053	0.887
Donor age, years±SD	51.38±10.27	50.37±10.95	0.095	0.480	51.38±10.27	51.98±10.47	−0.058	0.795
Graft weight, grams±SD	159.21±28.19	179.90±36.87	−0.631	<0.001	159.21±28.19	166.20±30.95	−0.236	0.211

ABOi, ABO incompatible; DM, diabetes mellitus; DSA+, donor specific antibody positive; HLA, human leucocyte antigen; IQR, Interquartile range; SMD, standardized mean difference.

### Intraoperative results

Intraoperative outcomes are presented in Table 2. RAKT, compared to OKT, was associated with significantly longer overall operation time (292.5 min vs. 230.0 min; *P*<0.001), in addition to longer RT (74.0 min vs. 36.0 min; *P*<0.001), NAT (27.0 min vs. 5.0 min; *P*<0.001), venous anastomosis time (24.0 min vs. 15.0 min; *P*<0.001), and arterial anastomosis time (20.5 min vs. 15.5 min; *P*<0.001).

**Table 2:** Intraoperative data.

	RAKT (*n*=50)	OKT (*n*=150)	*P*
Operation time, min (IQR)	292.50 (270.00–325.00)	230.00 (195.00–260.00)	<0.001
Console time, min (IQR)	134.00 (114.00–165.00)		
Open conversion, *n* (%)	2 (4.0)		
EBL, ml (IQR)	150.00 (100.00–250.00)	200.00 (100.00–300.00)	0.082
WIT, min (IQR)	2.00 (2.00–3.00)	3.00 (2.00–4.75)	<0.001
CIT, min (IQR)	57.50 (48.00–71.75)	49.00 (40.00–63.00)	0.012
RT, min (IQR)	74.00 (61.00–87.75)	36.00 (30.00–44.75)	<0.001
NAT, min (IQR)	27.00 (23.00–35.75)	5.00 (3.00–8.00)	<0.001
V anast time, min (IQR)	24.00 (19.00–29.00)	15.00 (12.00–19.00)	<0.001
A anast time, min (IQR)	20.50 (16.25–26.50)	15.50 (12.00–19.00)	<0.001
POD discharge, day (IQR)	11.00 (9.00–13.00)	10.00 (10.00–12.00)	0.632

CIT, cold ischemic time; EBL, estimated blood loss; IQR, Interquartile range; NAT, Nonanastomotic time; POD, postoperative day; RT, Rewarming Time; WIT, warm ischemic time.

Two robotic cases (4.0%) required open conversion due to abnormal perfusion. The open conversion was performed by extending the Pfannenstiel incision. Once arterial reanastomosis was done, improved renal perfusion was confirmed, and both cases were successfully completed.

### Surgical technique analysis

For the Shewhart control chart and CUSUM analysis, the upper cutoff of the target range was set as the corresponding OKT mean +1SD times: RT (52.6 min), NAT (14.4 min), venous anastomosis time (22.1 min), and arterial anastomosis time (22.7 min).

**Figure 3:**
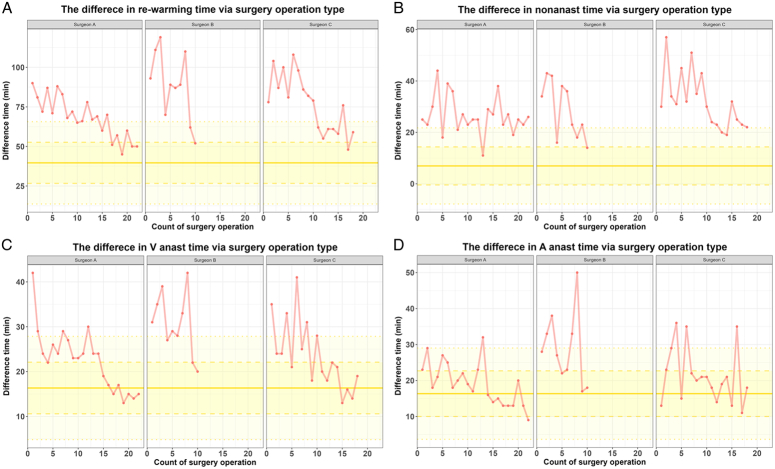
Shewhart control charts. (A) Rewarming Time, (B) Nonanastomosis time during RT, (C) Venous anastomosis time, (D) Arterial anastomosis time. Solid yellow line represents OKT mean time, dashed line represents OKT mean time +1SD (target value), and dotted line represents OKT mean time +2SD (alert threshold).

Intraoperative times and Shewhart control charts for each respective surgeon are depicted in Table 3 and Figure 3, respectively. RT was within target for (A: 18.2%, B: 10.0%, C: 5.6%), and below the alarm threshold for (A: 36.4%, B: 20.0%, C: 38.9%) of cases. NAT during RT met the target for (A: 4.6%, B: 10.0%, C: 0%), and below the alarm threshold for (A: 18.2%, B: 20.0%, C: 11.1%). Target was reached for venous anastomosis (A: 40.9%, B: 20.0%, C: 55.6%) and arterial anastomosis (A: 72.7%, B: 30.0%, C: 72.2%), and was below alarm threshold for venous anastomosis (A: 81.8%, B: 30.0%, C: 72.2%), and arterial anastomosis (A: 95.45%, B: 60.0%, C: 83.3%) in mentioned percentages.

**Table 3:** Intraoperative time distribution.

(A) Rewarming time	Within target (<52.6 min)	Target–alarm (52.6–65.6 min)	Above alarm (>65.6 min)
Surgeon A, *n* (%)	4 (18.2%)	4 (18.2%)	14 (63.6%)
Surgeon B, *n* (%)	1 (10.0%)	1 (10.0%)	8 (80.0%)
Surgeon C, *n* (%)	1 (5.6%)	6 (33.3%)	11 (61.1%)
Overall	6 (12.0%)	11 (22.0%)	33 (66.0%)
(B) Nonanastomotic time during RT	Within target (<14.4 min)	Target–alarm (14.4–21.7 min)	Above alarm (>21.7 min)
Surgeon A, *n* (%)	1 (4.6%)	3 (13.6%)	18 (81.8%)
Surgeon B, *n* (%)	1 (10.0%)	1 (10.0%)	8 (80.0%)
Surgeon C, *n* (%)	0 (0.0%)	2 (11.1%)	16 (88.9%)
Overall	2 (4.0%)	6 (12.0%)	42 (84.0%)
(C) Venous anastomosis time	Within target (<22.1 min)	Target–Alarm (22.1–27.8 min)	Above alarm (>27.8 min)
Surgeon A, *n* (%)	9 (40.9%)	9 (40.9%)	4 (18.2%)
Surgeon B, *n* (%)	2 (20.0%)	1 (10.0%)	7 (70.0%)
Surgeon C, *n* (%)	10 (55.6%)	3 (16.7%)	5 (27.8%)
Overall	21 (42.0%)	13 (26.0%)	16 (32.0%)
(D) Arterial anastomosis time	Within target (<22.7 min)	Target–alarm (22.7–29.0 min)	Above alarm (>29.0 min)
Surgeon A, *n* (%)	16 (72.7%)	5 (22.7%)	1 (4.6%)
Surgeon B, *n* (%)	3 (30.0%)	3 (30.0%)	4 (40.0%)
Surgeon C, *n* (%)	13 (72.2%)	2 (11.1%)	3 (16.7%)
Overall	32 (64.0%)	10 (20.0%)	8 (16.0%)

(A) Rewarming Time, (B) Nonanastomosis time during RT, (C) Venous anastomosis time, (D) Arterial anastomosis time.

Target time (OKT mean+1SD), Alarm threshold (OKT mean+2SD)

**Figure 4:**
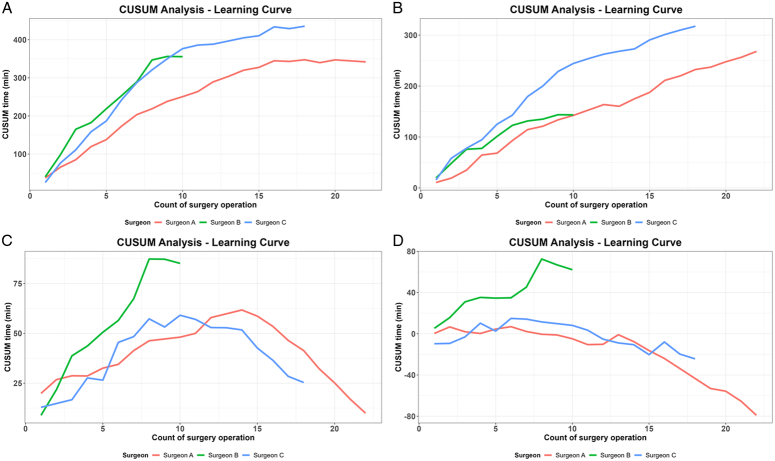
Cumulative summation analysis. (A) Rewarming Time, (B) Nonanastomosis time during RT, (C) Venous anastomosis time, (D) Arterial anastomosis time.

CUSUM analyses are displayed in Figure 4. Rewarming time learning curves for Surgeons A and C plateau at 15 cases, indicating its learning curve to be at least 15 cases. For NAT during RT, the learning curve has not yet been reached during the study period. The learning curve for venous and arterial anastomosis seems to require ~10 and 5 cases, respectively.

### Postoperative results

**Figure 5:**
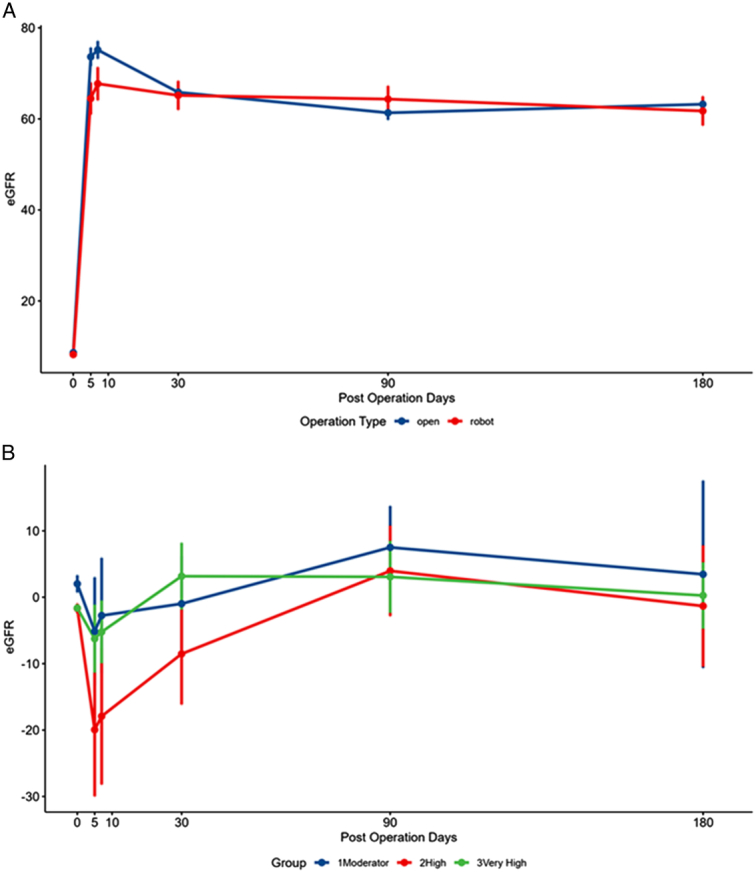
Postoperative graft function trend. (A) eGFR curve by operation type, (B) eGFR curve by risk group.

Postoperative graft function is reported in Table 4A and the overall eGFR trend is visualized in Figure 5A. RAKT patients exhibited lower eGFR values at POD 5 (64.48±24.93 vs. 73.63±24.96; *P*=0.026) and POD 7 (67.72±25.72 vs. 75.15±23.91; *P*=0.063). Graft function became increasingly comparable in later POD 1 month, 3 months, and 6 months follow-up periods. Evaluation of the overall eGFR trend using a mixed linear model indicated no significant difference between RAKT and OKT groups (51.35±2.64 vs. 54.01±1.45; *P*=0.315).

**Table 4:** Postoperative graft function.

(A) eGFR, ml/min/1.73 m^2^±SD	RAKT (*n*=50)	OKT (*n*=150)	*P*	
POD 0 days	8.24±3.79	8.65±3.18	0.462	
POD 5 days	64.48±24.93	73.63±24.96	0.026	
POD 7 days	67.72±25.72	75.15±23.91	0.063	
POD 1 months	65.16±22.26	65.85±19.41	0.836	
POD 3 months	64.34±19.05	61.34±18.89	0.361	
POD 6 months	61.74±19.52	63.22±18.33	0.669	
(B) eGFR, ml/min/1.73 m^2^±SD	Very high risk (*n*=21)	High risk (*n*=12)	Moderate risk (*n*=17)	*P*
POD 0 days	−1.6±2.5	−1.7±2.5	2.0±5.3	0.007
POD 5 days	−6.2±23.4	−20.0±34.7	−5.1±33.6	0.365
POD 7 days	−5.2±21.6	−17.9±35.6	−2.8±35.8	0.389
POD 1 months	3.2±22.5	−8.5±26.4	−1.0±27.7	0.453
POD 3 months	3.1±23.6	4.0±22.6	7.5±19.7	0.878
POD 6 months	0.3±18.7	−1.3±28.9	3.5±28.2	0.945

(A) eGFR by operation type, (B) Differences in eGFR between RAKT and matched OKT patients by risk group.

eGFR, estimated glomerular filtration rate, POD, postoperative day.

**Figure 6:**
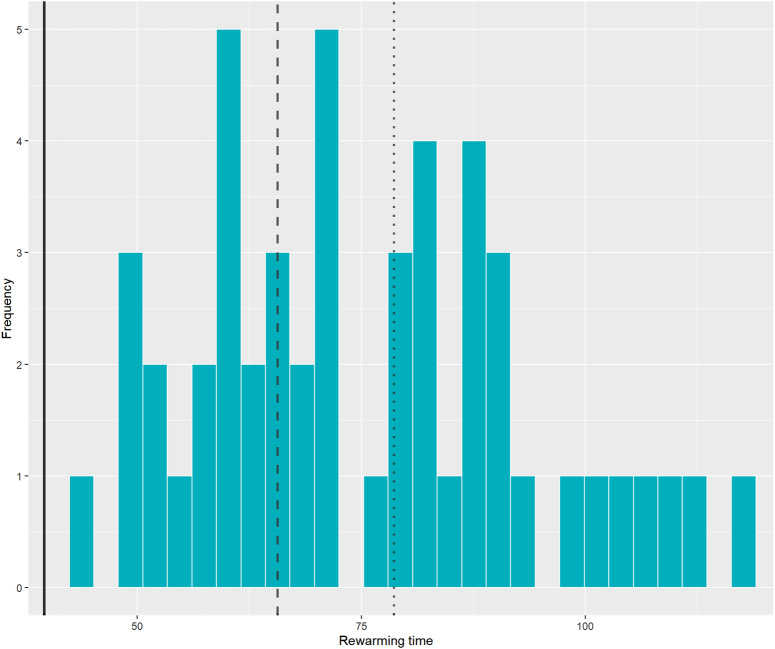
Density plot of RAKT rewarming times. Moderate risk (<OKT mean +2SD: <65.6 min), high risk (<OKT mean +2SD–3SD: 65.6 min–78.6 min), very high risk (>OKT mean +3SD: >78.6 min).

The differences in eGFR values between RAKT and corresponding matched OKT patients, and the overall trend compared across risk groups are shown in Table 4B and Figure 5B. As seen in Figure 6, RAKT patients were divided based on rewarming times, and classified into risk categories. Twenty-one patients were classified as very high risk (RT >78.6 min), 12 patients as high risk (RT 78.6 min to 65.6 min), and 17 patients as moderate risk (RT <65.6 min). Although not statistically significant, the high-risk group showed a notable decline in graft function at POD 5 and POD 7. This is likely attributed to outlier eGFR values from two DGF patients in this group. A mixed model analysis of the overall trend revealed no significant differences in eGFR between the very high-risk group (−2.66; *P*=0.694) and high-risk group (−9.23; *P*=0.238) when compared to the moderate-risk group (−1.08).

### Postoperative complications

There was no significant difference in SGF, DGF, graft survival, or patient survival. Three RAKT patients had SGF (6.0 vs. 2.67%, *P*=0.505), and DGF (6.0 vs. 1.3%, *P*=0.191). One RAKT case resulted in graft loss (2.0 vs. 1.33%, *P*=0.999), and no patient mortality was reported for both groups. The first patient, treated for recurred focal segmental glomerulosclerosis, underwent plasmapheresis and rituximab treatment. The second patient, diagnosed with antibody-mediated rejection, received plasmapheresis and immunoglobulin therapy. The last patient, exhibiting severe thrombotic microangiopathy, did not respond to plasmapheresis, ultimately resulting in graft nephrectomy at POD 36. The first two patients were part of the high risk, and the last patient was part of the moderate-risk group.

### Biopsy results

**Figure 7:**
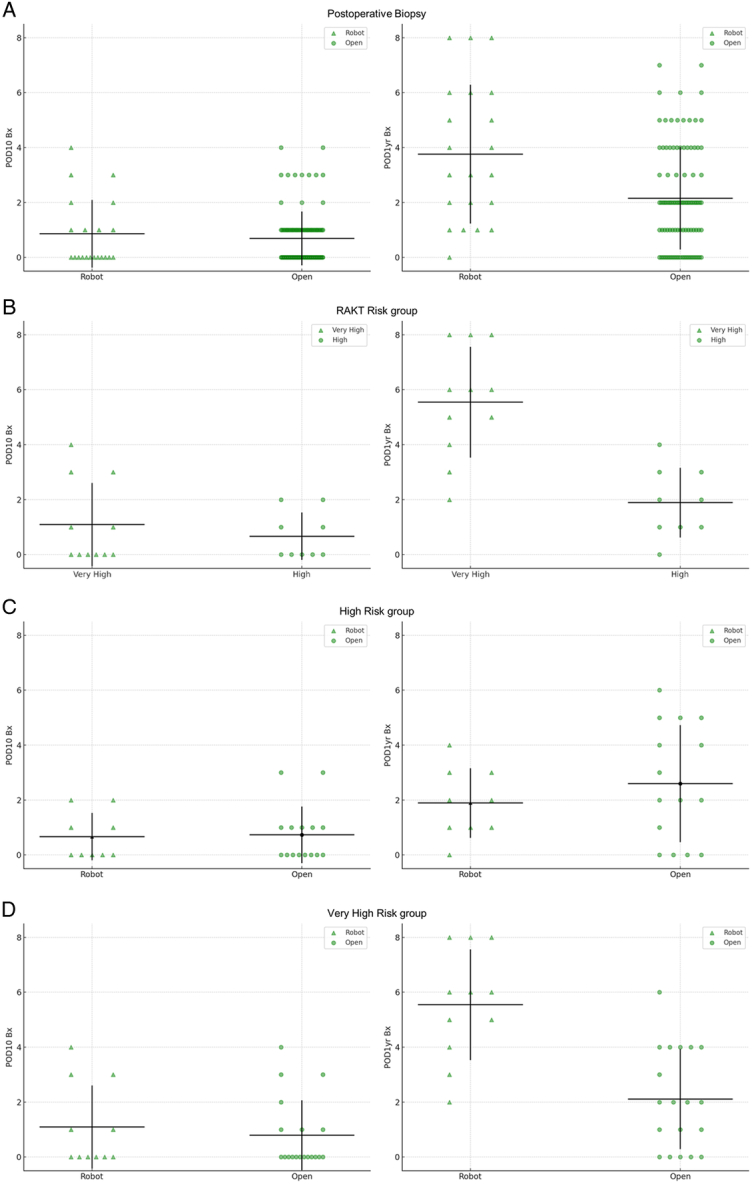
Postoperative Chronicity Index Score Scatterplot. (A) Chronicity Index scores by operation method, (B) Chronicity Index scores by RAKT risk groups, (C) Chronicity Index scores for high risk group, (D) Chronicity Index scores for very high risk group. Horizontal bar indicates mean and vertical bar indicates one SD.

Protocol biopsies conducted at POD 10 days and POD 1-year were analyzed. A total of 118 patients underwent both biopsies, and their demographics are detailed in Table 5. While weight and height differed significantly, there was no difference in BMI between the RAKT and OKT groups. Chronicity Index scores and Δ Chronicity Index, the difference between scores at POD 10 days and POD 1-year, were compared (Table 6A, Fig. 7A). Δ Chronicity Index was significantly greater in the RAKT group compared to the OKT group (2.91±1.92 vs. 1.46±1.76; *P*=0.002).

**Table 5:** Baseline characteristics of patients with biopsy results.

	Patients with biopsy		
	RAKT (*n*=21)	OKT (*n*=97)	*P*
ABOi, *n* (%)	5 (23.8)	22 (22.7)	1.000
DSA+, *n* (%)	0 (0.0)	8 (8.3)	0.377
Desensitization, *n* (%)	5 (23.8)	27 (27.8)	0.916
HLA mismatch count, *n*±SD	3.38±1.40	3.64±1.82	0.554
Recipient age, years±SD	33.81±12.61	37.65±13.33	0.205
Sex, M:F, *n* (%)	4 (19.1): 17 (81.0)	42 (43.3): 55 (56.7)	0.069
Weight, kg±SD	54.04±10.18	59.56±11.72	0.017
Height, cm±SD	160.65±9.63	164.97±7.56	0.043
BMI, kg/m^2^±SD	20.95±3.53	21.91±3.53	0.127
DM, *n* (%)	4 (19.1)	22 (22.7)	0.941
Dialysis, *n* (%)	19 (90.5)	68 (70.1)	0.099
Dialysis vintage, days (IQR)	341.0 (173.5–402.5)	183.5 (114.75–399.5)	0.350
Donor age, years±SD	50.86±10.70	50.96±10.84	0.860
Graft weight, grams±SD	156.07±27.72	161.90±29.67	0.379

ABOi, ABO incompatible; DM, diabetes mellitus; DSA+, donor specific antibody positive; HLA, human leucocyte antigen; IQR, interquartile range.

**Table 6:** Postoperative Chronicity Index Score.

(A) Chronicity Index	RAKT (*n*=21)	OKT (*n*=97)	*P*
POD 10 days	0.86±1.24	0.69±0.98	0.504
POD 1 years	3.76±2.53	2.16±1.87	0.001
Δ Chronicity Index	2.91±1.92	1.46±1.76	0.002
(B) Chronicity Index	Very high risk (*n*=11)	High risk (*n*=9)	*P*
POD 10 days	1.09±1.51	0.67±0.87	0.466
POD 1 years	5.55±2.02	1.89±1.27	<0.001
Δ Chronicity Index	4.45±1.92	1.22±1.48	<0.001
(C) Chronicity Index	High Risk (*n*=9)	Matched OKT (*n*=15)	*P*
POD 10 days	0.67±0.87	0.73±1.03	0.873
POD 1 years	1.89±1.27	2.60±2.13	0.376
Δ Chronicity Index	1.22±1.48	1.87±1.96	0.405
(D) Chronicity Index	Very high risk (*n*=11)	Matched OKT (*n*=9)	*P*
POD 10 days	1.09±1.51	0.79±1.27	0.564
POD 1 years	5.55±2.02	2.11±1.82	<0.001
Δ Chronicity Index	4.45±1.92	1.32±1.63	<0.001

(A) Chronicity Index scores by operation method, (B) Chronicity Index scores by RAKT risk groups, (C) Chronicity Index scores for high risk group, (D) Chronicity Index scores for very high risk group

Biopsy results were subsequently evaluated across RAKT risk categories (Table 6B, Fig. 7B). Results from the very high-risk (11 patients) and high-risk group (9 patients) were compared; the moderate-risk group was omitted as only one patient had complete data. The increase in Chronicity Index scores was considerably greater for the very high-risk group compared to the high-risk group (4.45±1.92 vs. 1.22±1.48; *P*<0.001).

Finally, biopsies of RAKT patients from the high risk (Table 6C, Fig. 7C) and very high risk (Table 6D, Fig. 7D) groups were compared to biopsies of corresponding matched OKT patients. Eleven OKT patients matched to high risk RAKT patients, and 19 OKT patients matched to very high risk RAKT patients had complete biopsy results. While the high risk group did not display any significant difference, Δ Chronicity Index for the very high risk group was significantly greater in the RAKT group (4.45±1.92 vs. 1.32±1.63, *P*<0.001).

## Discussion

In this study, we comprehensively outline our early experience with RAKT, describing our surgical technique, learning curve, and postoperative outcomes associated with RAKT. Our analysis suggests that the learning curve for rewarming time during RAKT requires at least 15 cases. Even during the early surgical experience, postoperative graft function and complications associated with kidney transplantation were comparable between the RAKT and OKT groups.

Although significantly longer rewarming times have been reported with RAKT, comparable long-term postoperative results seem to indicate its safety^31–33^. Furthermore, studies have shown the robotic method to be advantageous in decreasing postoperative pain and narcotic analgesic demand, in addition to reducing surgical site infections and hospital stay^34,35^. Even with these benefits, questions remain regarding postoperative outcomes during the initial experience of overcoming the learning curve, specifically for surgeons without prior experience in robotic surgery.

Our propensity score-matched cohort of 50 RAKT and 150 OKT patients resulted in similar findings to previous studies. We report significantly longer intraoperative times in the RAKT group. Early postoperative graft function (POD5, POD7) was marginally lower, but recovered to be comparable to OKT patients at POD 1 month, 3 months, and 6 months. Evaluation of overall eGFR trend using mixed linear model analysis also shows no significant difference in graft function. Further examination of patients classified into risk groups based on RT also reveals similar results.

With a growing emphasis on minimally invasive surgery and the development of the robotic surgical system, the implementation of robotic assistance for kidney transplantation was inevitable. Giolianotti *et al*.^8^ introduced the first fully robotic assisted kidney transplantation, and Menon *et al*.^25^ standardized the procedure that has been adopted by a number of centers. Our modifications include utilizing a Pfannenstiel incision for graft introduction and an open approach for ureteroneocystostomy. Given that our patient population predominantly comprises of Asian individuals with lower BMIs, the Pfannenstiel incision was a feasible choice. The incision was preferred for its cosmetic benefits, reduced wound pain and complications, and convenient access for quicker open ureteroneocystostomy^36,37^.

Previous studies on the RAKT learning curve were mostly limited to surgeons proficient in both OKT and robotic surgery. Ahlawat *et al*. reported mastery of rewarming time learning curve required 21 cases while the European Robotic Urological Society, a multicenter group, reported experienced surgeons required 35 cases to achieve acceptable rewarming time results^22,23^. It should also be noted, in the mentioned studies, target values derived from a reference group were used for CUSUM analysis. In another study, Sood *et al*.^21^ notably highlighted that surgeons lacking prior robotic experience faced significantly extended learning curves. This was of concern as all surgeons involved in this study, although experienced in OKT, lacked previous experience in robotic surgery.

Our analysis shows the learning curve for rewarming time during RAKT requires at least 15 cases, similar to a previous study involving a surgeon with no MIS experience^26^. Further analysis reveals relatively short vascular anastomosis learning curves of 5–10 cases, but an incomplete nonanastomotic time learning curve. Although Shewhart control charts for NAT indicate a generally improving trend, only two cases (4.0%) are within target and only eight cases (16.0%) are below the alarm threshold combined. Of note, a study involving surgeons skilled in both open KT and robotic surgeries reported that 62.9% of cases fell below the NAT alarm threshold of 14.8 min^23^. Even with our higher NAT alarm threshold of 21.7 min, a noticeably lower percentage of cases fell below the alarm threshold.

While robotic vascular anastomosis, predominantly performed individually by the operating surgeon, quickly improved, the nonanastomotic phase, primarily consisting of graft positioning and collaborative work with the surgical team, has been a challenge. Surgeons were likely able to leverage their background in open vascular anastomosis with the inherent benefits of the robotic system, such as high magnification, tremor elimination, and greater range of motion, to improve their robotic vascular anastomosis times^38^. Longer NATs may be attributed to operating surgeons’ early experience with robotic surgery. Even though surgeons in our group completed Intuitive’s training program, practiced robotic vascular anastomosis, and held multiple planning meetings, additional cadaveric or animal workshops may be necessary for adequate preparation, as further coordination within the surgical team may help improve operative efficiency. Although it may be unrealistic to expect NAT of RAKT to be comparable to that of OKT, poor robotic NATs highlight the importance of establishing efficient surgical teamwork, as NAT appears to be the most improvable aspect during rewarming time.

Our study is unique in our evaluation of postoperative biopsies involving RAKT patients. Histological evaluation of POD 10 day and POD 1-year protocol biopsies shows a significant difference in Δ Chronicity Index scores of RAKT patients compared to those of OKT patients. Furthermore, the very high risk group, composed of patients with the longest RTs, displays significantly greater Δ Chronicity Index scores compared to both the high risk group and their corresponding OKT counterparts. Longer rewarming times during RAKT seem to be associated with chronic histological damage to graft kidneys. On the other hand, as evidenced by the low Δ Chronicity Index scores in the high risk group, there is optimism that improving rewarming times may help mitigate histological injury.

It is known that longer ischemic times are associated with poor graft function and histological injury, and chronicity scores can be predictive of loss of kidney function^39–42^. Fortunately, in our study, overall graft function is similar between RAKT and OKT groups, as well as across RAKT risk groups. In addition, postoperative complications rates, including DGF and graft survival, are comparable across both groups. This may be reassuring for surgeons implementing a new RAKT program; however, as seen by the difference in Δ Chronicity Index scores, chronic histological damage associated with long RT in RAKT patients indicates careful long-term follow-up is necessary.

This study comprehensively examines our initial experience of RAKT, providing a detailed analysis of the learning curve and postoperative outcomes. We analyzed the learning curves of three separate surgeons, all with no MIS experience, offering valuable insight for surgeons with no such experience. To the best of our knowledge, this is the first study to incorporate postoperative histological evaluations in the context of RAKT. Our evaluation of protocol biopsies enhances our understanding of how the novel RAKT procedure may affect graft kidneys.

Major limitations of this study include its nonrandomized, retrospective nature. To best minimize potential biases, propensity score matching was done, achieving a high degree of similarity. As our study is a single-center study, generalizability may be limited, and a multicenter study may be helpful. Additionally, our study population consists of Asian patients with a lower BMI distribution, and all grafts were from living donors. Lastly, considering the concerning histological results, a 6-month follow-up period may not be enough, and long-term graft function must also be evaluated.

## Conclusions

Our findings contribute to the growing body of literature, emphasizing the feasibility and safety of RAKT. Even among surgeons with no prior robotic experience, significant differences in graft function or postoperative complications were not observed during their early experience. However, analysis of postoperative protocol biopsies revealed chronic histological injury, underscoring the need for vigilant patient follow-up.

Intraoperative analysis suggests at least 15 cases are required to overcome the learning curve for rewarming time. Given the relatively short learning curve for vascular anastomoses, reducing nonanastomotic time during rewarming time appears to be critical, highlighting the importance of establishing efficient collaboration within the surgical team.

Based on current findings and ongoing advancements in robotic surgery, RAKT shows promise to become a standard procedure for kidney transplantation. To realize this potential, further refinement of operative protocols to enhance surgical efficiency and research focusing on long-term outcomes to ensure sustained benefits for RAKT recipients will be critical.

## Ethical approval

Seoul National University Hospital Institutional Review Board (IRB No 2305-119-1434).

## Consent

Not applicable.

## Source of funding

This research was supported by a grant of the Korea Health Technology R&D Project through the Korea Health Industry Development Institute (KHIDI), funded by the Ministry of Health and Welfare, Republic of Korea (grant number: HI23C1591).

## Author contribution

S.J.J.K., S.M., and A.H.: study concept, design, data collection, data analysis and interpretation, and writing the paper; S.K.: data analysis and interpretation; A.C. and J.H.: study concept and design.

## Conflicts of interest disclosure

The authors declare no conflicts of interest.

## Research registration unique identifying number (UIN)

Registered on clinicaltrials.gov (NCT 06320821).

## Guarantor

Sangil Min, MD, PhD, Department of Surgery, Seoul National University College of Medicine, Seoul, Republic of Korea. Tel.: +82 2072 2330. E-mail: surgeonmsi@gmail.com.

## Data availability statement

The journal requires authors to include in any articles that report results derived from research data to include a data availability statement. Please confirm if any datasets generated during and/or analyzed during the current study are publicly available, available upon reasonable request, or if data sharing is not applicable to this article.

## Provenance and peer review

Not applicable.

## References

[1] Collins AJ Foley RN Gilbertson DT . United States Renal Data System public health surveillance of chronic kidney disease and end-stage renal disease. Kidney Int Suppl (2011) 2015;5:2–7. 26097778 10.1038/kisup.2015.2 PMC4455192

[2] Merrill J Murray J Harrison H . Successful homotransplantation of the human kidney between identical twins. J Am Med Associat 1956;160:277–282. 10.1001/jama.1956.02960390027008 13278189

[3] Gandaglia G Ghani KR Sood A . Effect of minimally invasive surgery on the risk for surgical site infections: results from the National Surgical Quality Improvement Program (NSQIP) Database. JAMA Surg 2014;149:1039–1044. 25143176 10.1001/jamasurg.2014.292

[4] Coccolini F Catena F Pisano M . Open versus laparoscopic cholecystectomy in acute cholecystitis. Systematic review and meta-analysis. Int J Surg 2015;18:196–204. 25958296 10.1016/j.ijsu.2015.04.083

[5] Corcione F Esposito C Cuccurullo D . Advantages and limits of robot-assisted laparoscopic surgery: preliminary experience. Surg Endosc 2005;19:117–119. 15549629 10.1007/s00464-004-9004-9

[6] Gachabayov M Lee H Kajmolli A . Impact of robotic total mesorectal excision upon pathology metrics in overweight males with low rectal cancer: a pooled analysis of 836 cases. Updates Surg 2024;76:505–512. 38147292 10.1007/s13304-023-01733-y

[7] Shadmanov N Aliyev V Piozzi GN . Perioperative and long-term oncological outcomes of robotic versus laparoscopic total mesorectal excision: a retrospective study of 672 patients. J Robot Surg 2024;18:144. 38554211 10.1007/s11701-024-01922-w

[8] Giulianotti P Gorodner V Sbrana F . Robotic transabdominal kidney transplantation in a morbidly obese patient. Am J Transplant 2010;10:1478–1482. 20486912 10.1111/j.1600-6143.2010.03116.x

[9] Breda A Territo A Gausa L . Robot-assisted kidney transplantation: the European Experience. Eur Urol 2018;73:273–281. 28916408 10.1016/j.eururo.2017.08.028

[10] Lee SD Rawashdeh B McCracken EKE . Robot-assisted kidney transplantation is a safe alternative approach for morbidly obese patients with end-stage renal disease. Int J Med Robot 2021;17:e2293. 34080270 10.1002/rcs.2293

[11] Oberholzer J Giulianotti P Danielson KK . Minimally invasive robotic kidney transplantation for obese patients previously denied access to transplantation. Am J Transplant 2013;13:721–728. 23437881 10.1111/ajt.12078 PMC3647345

[12] Kim HJ Yang SJ Jeong W . The first robotic kidney transplantation in Korea: a case report. Korean J Transplant 2022;36:61–66. 35769429 10.4285/kjt.21.0023 PMC9235531

[13] Lim SJ Ko Y Kim DH . Robot-assisted kidney transplantation. JoVE 2021;173:e62220. 10.3791/62220 34338681

[14] Fan Y Zhao J Zu Q . Robot-assisted kidney transplantation: initial experience with a modified hypothermia technique. Urol Int 2022;106:504–511. 35152213 10.1159/000521959

[15] Tzvetanov I D’Amico G Benedetti E . Robotic-assisted kidney transplantation: our experience and literature review. Curr Transplant Rep 2015;2:122–126. 26000230 10.1007/s40472-015-0051-z PMC4431703

[16] Spaggiari M Lendacki FR Di Bella C . Minimally invasive, robot-assisted procedure for kidney transplantation among morbidly obese: positive outcomes at 5 years post-transplant. Clin Transplant 2018;32:e13404. 30216555 10.1111/ctr.13404

[17] Kishore TA Kuriakose MJ Pathrose G . Robotic assisted kidney transplantation in grafts with multiple vessels: single center experience. Int Urol Nephrol 2020;52:247–252. 31586280 10.1007/s11255-019-02305-z

[18] Maheshwari R Qadri SY Rakhul LR . Prospective nonrandomized comparison between open and robot-assisted kidney transplantation: analysis of midterm functional outcomes. J Endourol 2020;34:939–945. 32600060 10.1089/end.2020.0213

[19] Musquera M Peri L Ajami T . Results and lessons learned on robotic assisted kidney transplantation. Biomed Res Int 2020;2020:8687907. 32934965 10.1155/2020/8687907 PMC7484686

[20] Pein U Girndt M Markau S . Minimally invasive robotic versus conventional open living donor kidney transplantation. World J Urol 2020;38:795–802. 31127330 10.1007/s00345-019-02814-7

[21] Sood A Ghani KR Ahlawat R . Application of the statistical process control method for prospective patient safety monitoring during the learning phase: robotic kidney transplantation with regional hypothermia (IDEAL phase 2a-b). Eur Urol 2014;66:371–378. 24631408 10.1016/j.eururo.2014.02.055

[22] Ahlawat RK Tugcu V Arora S . Learning curves and timing of surgical trials: robotic kidney transplantation with regional hypothermia. J Endourol 2018;32:1160–1165. 29587531 10.1089/end.2017.0697

[23] Gallioli A Territo A Boissier R . Learning curve in robot-assisted kidney transplantation: results from the European Robotic Urological Society Working Group. Eur Urol 2020;78:239–247. 31928760 10.1016/j.eururo.2019.12.008

[24] Mathew G Agha R Group S . STROCSS 2021: strengthening the reporting of cohort, cross-sectional and case-control studies in surgery. Ann Med Surg (Lond) 2021;72:103026. 34820121 10.1016/j.amsu.2021.103026 PMC8599107

[25] Menon M Sood A Bhandari M . Robotic kidney transplantation with regional hypothermia: a step-by-step description of the Vattikuti Urology Institute-Medanta technique (IDEAL phase 2a). Eur Urol 2014;65:991–1000. 24388099 10.1016/j.eururo.2013.12.006

[26] Kim HJ Jeong W Lee J . Successful robotic kidney transplantation for surgeons with no experience in minimally invasive surgery: a single institution experience. Int J Surg 2024;110:1586–1594. 38052024 10.1097/JS9.0000000000000977 PMC10942182

[27] Inker LA Eneanya ND Coresh J . New creatinine- and cystatin c-based equations to estimate GFR without race. N Engl J Med 2021;385:1737–1749. 34554658 10.1056/NEJMoa2102953 PMC8822996

[28] Haas M Loupy A Lefaucheur C . The Banff 2017 Kidney Meeting Report: revised diagnostic criteria for chronic active T cell-mediated rejection, antibody-mediated rejection, and prospects for integrative endpoints for next-generation clinical trials. Am J Transplant 2018;18:293–307. 29243394 10.1111/ajt.14625 PMC5817248

[29] Loupy A Haas M Roufosse C . The Banff 2019 Kidney Meeting Report (I): Updates on and clarification of criteria for T cell- and antibody-mediated rejection. Am J Transplant 2020;20:2318–2331. 32463180 10.1111/ajt.15898 PMC7496245

[30] Haas M Mirocha J Huang E . A Banff-based histologic chronicity index is associated with graft loss in patients with a kidney transplant and antibody-mediated rejection. Kidney Int 2023;103:187–195. 36332728 10.1016/j.kint.2022.09.030 PMC11466365

[31] Kishore TA Kaddu DJ Sodhi BS . Robotic kidney transplant beyond the learning curve: 8-year single-center experience and matched comparison with open kidney transplant. Urology 2024;183:100–105. 37952604 10.1016/j.urology.2023.10.031

[32] Patil A Ganpule A Singh A . Robot-assisted versus conventional open kidney transplantation: a propensity matched comparison with median follow-up of 5 years. Am J Clin Exp Urol 2023;11:168–176. 37168935 PMC10165225

[33] Tzvetanov IG Spaggiari M Tulla KA . Robotic kidney transplantation in the obese patient: 10-year experience from a single center. Am J Transplant 2020;20:430–440. 31571369 10.1111/ajt.15626

[34] Ahlawat R Sood A Jeong W . Robotic kidney transplantation with regional hypothermia versus open kidney transplantation for patients with end stage renal disease: an ideal stage 2B study. J Urol 2021;205:595–602. 32941100 10.1097/JU.0000000000001368

[35] Slagter JS Outmani L Tran K . Robot-assisted kidney transplantation as a minimally invasive approach for kidney transplant recipients: a systematic review and meta-analyses. Int J Surg 2022;99:106264. 35183735 10.1016/j.ijsu.2022.106264

[36] Luijendijk R Jeekel J Storm R . The low transverse pfannenstiel incision and the prevalence of incisional hernia and nerve entrapment. Ann Surg 1997;225:365–369. 9114794 10.1097/00000658-199704000-00004 PMC1190743

[37] Gizzo S Andrisani A Noventa M . Caesarean section: could different transverse abdominal incision techniques influence postpartum pain and subsequent quality of life? A systematic review. PLoS One 2015;10:e0114190. 25646621 10.1371/journal.pone.0114190 PMC4315586

[38] Diana M Marescaux J . Robotic surgery. Br J Surg 2015;102:e15–e28. 25627128 10.1002/bjs.9711

[39] Srivastava A Palsson R Kaze AD . The prognostic value of histopathologic lesions in native kidney biopsy specimens: results from the boston kidney biopsy cohort study. J Am Soc Nephrol 2018;29:2213–2224. 29866798 10.1681/ASN.2017121260 PMC6065095

[40] Tennankore KK Kim SJ Alwayn IP . Prolonged warm ischemia time is associated with graft failure and mortality after kidney transplantation. Kidney Int 2016;89:648–658. 26880458 10.1016/j.kint.2015.09.002

[41] Urbanellis P Mazilescu L Kollmann D . Prolonged warm ischemia time leads to severe renal dysfunction of donation-after-cardiac death kidney grafts. Sci Rep 2021;11:17930. 34504136 10.1038/s41598-021-97078-w PMC8429572

[42] Wickham J . Regional renal hypothermia. Ann Royal Coll Surg England 1971;48:99–113. PMC2387812 5551195

